# Human multidrug resistance protein 4 (MRP4) is a cellular efflux transporter for paracetamol glutathione and cysteine conjugates

**DOI:** 10.1007/s00204-020-02793-4

**Published:** 2020-05-29

**Authors:** Jan B. Koenderink, Jeroen J. M. W. van den Heuvel, Ab Bilos, Galvin Vredenburg, Nico P. E. Vermeulen, Frans G. M. Russel

**Affiliations:** 1 Department of Pharmacology and Toxicology, Radboud University Medical Center, Radboud Institute for Molecular Life Sciences (RIMLS), Nijmegen, The Netherlands; 2 Division of Molecular Toxicology, Department of Chemistry and Pharmaceutical Sciences, VU University Amsterdam, Amsterdam, The Netherlands

**Keywords:** Multidrug Resistance Protein 4 (MRP4), Paracetamol, Acetaminophen (APAP), 3-Cysteinyl-acetaminophen trifluoroacetic acid salt (APAP-CYS), Acetaminophen-glutathione (APAP-SG), Membrane vesicles, Transport

## Abstract

Paracetamol (acetaminophen, APAP) overdose is a leading cause of acute drug-induced liver failure. APAP hepatotoxicity is mediated by the reactive metabolite N-acetyl-*p*-benzoquinone imine (NAPQI). NAPQI is inactivated by conjugation with glutathione (GSH) to APAP-GSH, which is further converted into its cysteine derivative APAP-CYS. Before necrosis of hepatocytes occurs, APAP-CYS is measurable in plasma of the affected patient and it has been proposed as an early biomarker of acetaminophen toxicity. APAP-GSH and APAP-CYS can be extruded by hepatocytes, but the transporters involved are unknown. In this study we examined whether ATP-binding cassette (ABC) transporters play a role in the cellular efflux of APAP, APAP-GSH, and APAP-CYS. The ABC transport proteins P-gp/ABCB1, BSEP/ABCB11, BCRP/ABCG2, and MRP/ABCC1-5 were overexpressed in HEK293 cells and membrane vesicles were produced. Whereas P-gp, BSEP, MRP3, MRP5, and BCRP did not transport any of the compounds, uptake of APAP-GSH was found for MRP1, MRP2 and MRP4. APAP-CYS appeared to be a substrate of MRP4 and none of the ABC proteins transported APAP. The results suggest that the NAPQI metabolite APAP-CYS can be excreted into plasma by MRP4, where it could be a useful biomarker for APAP exposure and toxicity. Characterization of the cellular efflux of APAP-CYS is important for its development as a biomarker, because plasma concentrations might be influenced by drug-transporter interactions and upregulation of MRP4.

**Electronic supplementary material:**

The online version of this article (10.1007/s00204-020-02793-4) contains supplementary material, which is available to authorized users.

## Introduction

Paracetamol (N-acetyl-para-aminophenol, APAP or acetaminophen) is the most used over-the-counter pain reliever and fever reducer worldwide. It is a safe drug with minor side effects, but when overdosed it causes acute liver failure (McGill and Jaeschke 2013). At therapeutic dose the half-life of paracetamol is 1.5–3 h, it is glucuronidated (50–70%) and sulfated (25–35%) in the liver, after which it is secreted in the urine. A small percentage of paracetamol is catalyzed by cytochrome P450 enzymes into the reactive metabolite N-acetyl-p-benzoquinone imine (NAPQI). Detoxification of NAPQI occurs both spontaneously and enzymatically through its binding to the cysteine thiol of glutathione (GSH) to form APAP-GSH, which is further converted to its cysteine derivative or excreted into bile and plasma (Mazaleuskaya et al. 2015). An overdose of paracetamol results in the formation of excess NAPQI, which can deplete GSH levels and result in hepatocyte necrosis due to protein binding and mitochondrial damage (McGill and Jaeschke 2013).

Cysteine adducts of NAPQI have been detected in serum during paracetamol hepatotoxicity. Release of paracetamol adducts with therapeutic doses occurs in the absence of cell lysis (McGill et al. 2011, 2013) and serum APAP-CYS concentrations were reported to remain below 1 μM (Heard et al. 2011). However, when paracetamol is overdosed and hepatic necrosis occurs, adducts are released as a result of hepatocyte lysis and reach values up to 30 μM (Curry et al. 2019). Several studies have shown that serum APAP-CYS could be a useful diagnostic marker for paracetamol overdose in cases of liver injury of unknown or uncertain cause (Curry et al. 2019). The long half-life of NAPQI adducts (1–2 days) compared to the short serum half-life of paracetamol is making this a much better option than determining the serum paracetamol concentrations (Curry et al. 2019).

Murine studies showed that biliary excretion of both APAP-glucuronide and APAP-sulfate is largely dependent on Mrp2 and Bcrp activity (Chen et al. 2003; Lee et al. 2009; Xiong et al. 2000, 2002; Zamek-Gliszczynski et al. 2006), whereas basolateral excretion of APAP-glucuronide is mediated by Mrp3 and of APAP-sulfate by Mrp3 and Mrp4 (Manautou et al. 2005; Zamek-Gliszczynski et al. 2006). In addition, in humans the role of MRPs and BCRP for APAP-sulfate transport was indicated by inhibition studies with the BeWo cell line (Mitra and Audus 2010).

The involvement of Mrp2 in the biliary excretion of APAP-GSH is supported by the finding that in transport-deficient (TR-) rats APAP-GSH was virtually absent from the bile, whereas APAP-CYS was not affected (Chen et al. 2003). The transporter(s) responsible for the efflux of APAP-CYS are unknown. In this study we used membrane vesicles of HEK293 cells overexpressing the human apical hepatocyte ABC transport proteins P-gp/ABCB1, BSEP/ABCB11, MRP2/ABCC2, and, BCRP/ABCG2, and the basolateral transporters MRP1/ABBC1, MRP3/ABCC3, MRP4/ABCC4 and MRP5/ABCC5 to determine which transporters are involved in excretion of APAP-GSH and APAP-CYS from the hepatocyte at therapeutic doses.

## Methods

### Materials

Gateway^**®**^ cloning system was from ThermoFisher Scientific (Waltham, Massachusetts, USA). [^3^H]-methotrexate ([^3^H]-MTX) was purchased from Moravek Biochemicals (Brea, California, USA). Acetaminophen (APAP), adenosine 5′-triphosphate disodium salt (ATP) (from bacterial source), adenosine 5′-monophosphate monohydrate (AMP) (from yeast) were purchased from Sigma-Aldrich (Saint Louis, Missouri, USA). 3-Cysteinylacetaminophen trifluoroacetic acid salt (APAP-CYS) was from Santa Cruz Biotechnology (Dallas, Texas, USA). Acetaminophen-glutathione (APAP-GSH) was synthesized as previously described (Vredenburg et al. 2014). Multiscreen_HTS_ filter plates and Vacuum Manifold filtration device were from Merck-Millipore (Burlington, Massachusetts, USA). The LC–MSMS system consisted of a HPLC (Accela^®^), a quaternary ultrahigh-pressure pump, a vacuum degasser and an autosampler (TSQ Vantage^®^) coupled to a triple quadrupole mass spectrometer and were from ThermoFisher Scientific (Waltham, Massachusetts, USA).

### Production of transport proteins in membrane vesicles

The ABC transport proteins P-gp/ABCB1, BSEP/ABCB11, BCRP/ABCG2, and MRP/ABCC1-5 were overexpressed in HEK293 cells using a mammalian baculovirus system. The ABCB11 gene coding for BSEP was cloned into recombinant baculovirus and BSEP activity was tested with tauro[carbonyl-^3^H]cholic acid as described before (van Beusekom et al. 2013). A PCR product containing the ABCC5 gene with accession number NM_005688 was derived from kidney cDNA and cloned into the pDONR201 vector with the gateway system to obtain the pENTR-hMRP5 vector. With the Gateway^**®**^ LR reaction the expression vector BacMam-hMRP5 was generated. The production of recombinant MRP5 virus and subsequent transduction of HEK293 cells was performed as described previously (Wittgen et al. 2011). The activity of the MRP5 vesicles was tested with [^3^H]-methotrexate as a substrate (Lempers et al. 2016). HEK293 membrane vesicles overexpressing MRP1-4, BCRP, P-gp, or Ctrl (eYFP protein) were isolated and functionally tested with [6,7-^3^H(*N*)]Estradiol 17-β-d-glucuronide, [^3^H]-estrone sulphate, and [^3^H]N-methyl-quinidine respectively, as previously described (Dankers et al. 2012; Wittgen et al. 2011, 2012).

### Transport assay for APAP, APAP-GSH and APAP-CYS

Membrane vesicles isolated from ABC transporter-expressing HEK cells consist of a mixture of inside-out and rightside-out membrane vesicles. Only the inside-out-oriented vesicle fraction reacts with ATP to transport substrates into the vesicle. Values obtained for ATP-dependent transport affinities (Km values) are not affected by the percentage of inside-out-vesicles (Brouwer et al. 2013). Uptake of APAP, APAP-GSH and APAP-CYS by membrane vesicles (7.5 µg) containing MRP1-5, BCRP, P-gp, or BSEP was tested by incubation for 5 min at 37 °C in TS buffer (10 mM Tris-HEPES, pH 7.4; 250 mM Sucrose) supplemented with 10 mM MgCl_2_, 4 mM AMP or ATP and 0.5 mM APAP, APAP-GSH or APAP-CYS. Transport was stopped by placing the mixture on ice, after which it was filtered on a PVDF filter plate with a vacuum manifold filtration device and washed twice with ice-cold TS buffer. APAP, APAP-GSH, and APAP-CYS was extracted from the vesicles on the filter with 20% acetonitrile. By changing the incubation time and the compound concentration, time curves, and Michaelis–Menten plots were generated for the compound-transporter combinations that showed uptake activity. Samples were measured with LC-MS/MS.

### Chromatographic and mass spectrometric conditions

Liquid Chromatographic (LC) separation was performed using an HSS T3 analytical column (1.8 μm; 100 × 2.1 mm, Acquity UPLC^®^, Waters, Ireland) coupled with a VanGuard^®^ HSS T3 pre-column (1.8 µm; 5 × 2.1 mm, Acquity UPLC^®^, Waters, Ireland). The mobile phase (flow 350 µL/min) consisted of solvent A (0.1% (v/v) formic acid (HCOOH) in water) at 0, 0.5, 4.0 and 10 min and 15% A and 85% solvent B (0.1% (v/v) formic acid (HCOOH) in acetonitrile) at 3.5 min.

Chromatography was performed at an oven temperature of 30 °C. The sample injection volume was 10 µL and the analysis run time 10 min. The samples were stored at a tray temperature of 8 °C.

The compound-dependent parameters were optimized for the target compounds APAP, APAP-SG, and APAP-Cys to achieve the highest instrument response. MS parameters using positive ion mode were optimized for achieving good sensitivity for all compounds in one single analytical run, and operating conditions by direct infusion of a 1 µM mixture of both analytes.

Heated electrospray ionization (HESI) was operated at a spray voltage of + 3.5 kV, a capillary temperature of 239 °C, and a vaporizer temperature of 382 °C. Nitrogen was used as sheath and auxiliary gas with a gas pressure of 20 and 15 AU (Arbitrary Units), respectively. Argon was used as collision gas at a pressure of 1.5 mTorr. During the LC–HESI–MS/MS analysis, a time-segment program was developed to switch the divert valve of the mobile phase to waste or detection mode to prevent ion suppression and contamination of the ion source.

Positive ion mode was used with selected reaction monitoring (SRM) for the quantitative analysis of APAP, APAP-SG and APAP-Cys. The most abundant product ion was used for quantification, which was performed using peak areas. A second product ion was used for qualification purposes. The optimal SRM transitions and collision energies (CE) were determined as shown in Table 1.

**Table 1:** SRM Transitions and Collision Energies

Compound name	Precursor ion (m/z)	Product ion (m/z)	CE (eV)
APAP	152.06	110.04	15
APAP qualifier	152.06	65.03	33
APAP-SG	457.09	139.96	37
APAP-SG qualifier	457.09	328.08	16
APAP-Cys	271.10	140.00	25
APAP-Cys qualifier	271.10	96.00	42

### Data analysis

Prism software, version 5.03 (GraphPad Software Inc., San Diego, CA) was used for data analysis, curve fitting (Michaelis–Menten), K_m_ determination, and statistical analysis. The paired *t* test was used to determine significant ATP-dependent transport (*p* < 0.05). ATP-dependent transport data is presented as mean ± S.E.M. and the K_m_ is presented as mean ± S.E.

## Results

### Studying APAP, APAP-GSH and APAP-CYS as possible substrates

**Fig. 1:**
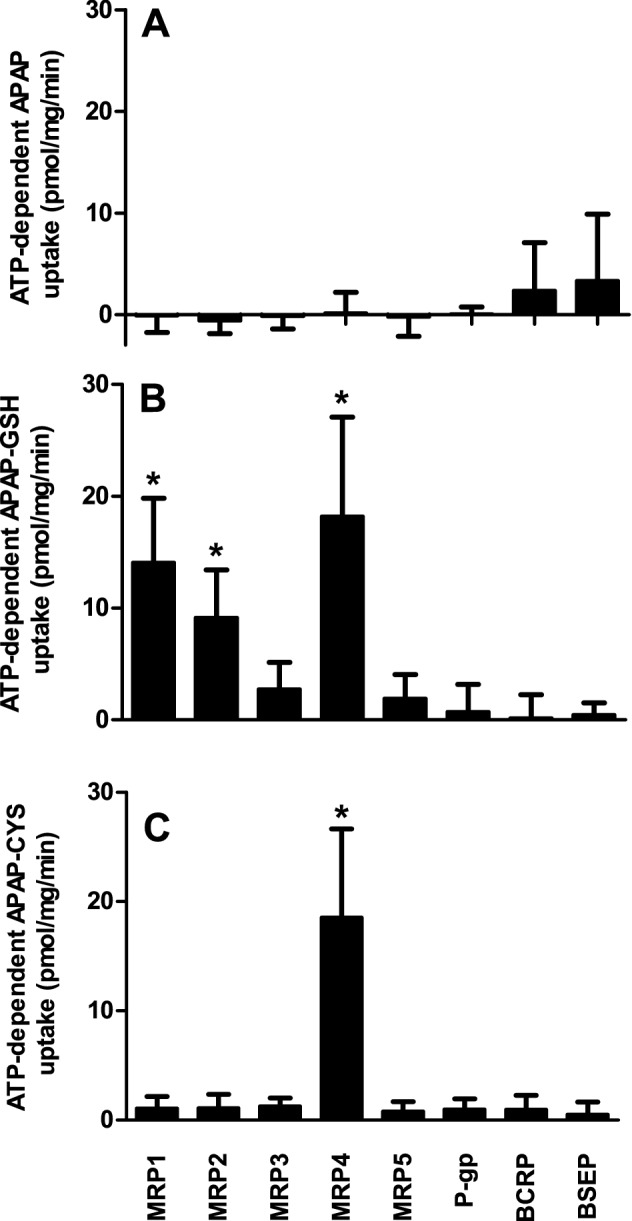
P-gp, BSEP, MRP1-5, and BCRP transport of APAP and its conjugates APAP-GSH and APAP-CYS. ATP-dependent uptake of 500 µM APAP **a**, APAP-GSH **b** or APAP-CYS **c** by P-gp, BSEP, MRP1-5 and BCRP (7.5 µg protein) was measured during 5 min incubation at 37 °C. Values are presented as mean ± SEM with *n* = 5–6. *p < 0.05

The vesicular transport assay was used to determine if APAP, APAP-GSH, and APAP-CYS are transport substrates of MRP1-5, BCRP, P-gp or BSEP. The ATP-dependent uptake of model compounds into the membrane vesicles demonstrated that these transporters were active (see Methods). None of the tested transporters gave ATP-dependent uptake of APAP (*p* < 0.05, Fig. 1a). APAP-GSH showed ATP-dependent transport with MRP1, MRP2, and MRP4 (Fig. 1b), and APAP-CYS was only transported by MRP4 (Fig. 1c).

### Kinetics of APAP-GSH and APAP-CYS transport

**Fig. 2:**
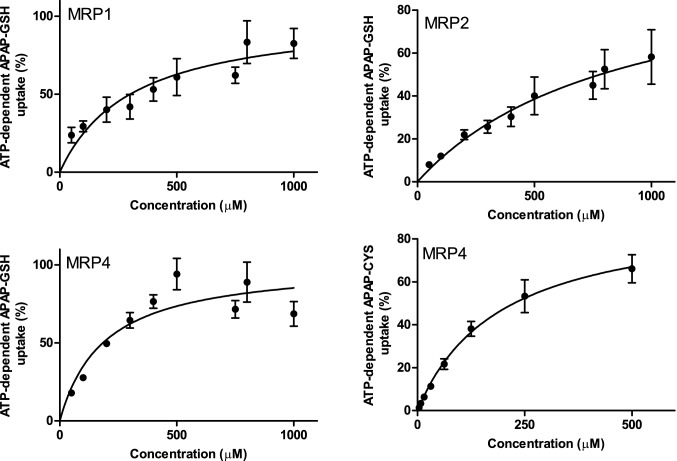
Concentration-dependent transport of APAP-GSH or APAP-CYS by MRP1, MRP2, and MRP4. Measurements were performed with 50–500 µM APAP-GSH or APAP-CYS for 5 min at 37 °C. Values are presented as mean ± SEM with n = 4–8

Kinetic parameters were determined for the transporters that showed activity with one of the APAP conjugates. Linear uptake up to 20 min was observed for MRP1, MRP2 and MRP4 using APAP-GSH as substrate (Additional file 1: Fig. S1). The APAP-CYS time curve of MRP4 was linear up to 10 min. To ensure initial transport rate condition for proper kinetic analysis, concentration-dependent uptake was measured after 5 min incubation. Transport by all transporters followed simple Michaelis–Menten kinetics resulting in *K*_m_ values for APAP-GSH by MRP1, MRP2 and MRP4 of 310 ± 70, 880 ± 240 and 190 ± 40 µM, respectively (Fig. 2), and for APAP-CYS by MRP4 of 190 ± 50 µM (mean ± S.E.).

## Discussion

Paracetamol (APAP) intoxication is a frequent cause of drug-induced liver injury. To discern other causes of liver injury from APAP-induced failure the metabolite APAP-CYS has been suggested as a plasma biomarker (McGill and Jaeschke 2013). The mechanism by which APAP-CYS is effluxed from liver cells has not been revealed yet. In this study we show that the sinusoidal membrane transporter MRP4 is able to actively translocate APAP-CYS.

Paracetamol is a safe drug and only after overdosing it will induce liver toxicity due to adduct formation. At therapeutic concentrations most of the APAP is eliminated as glucuronide and sulfate conjugates, but a minor amount is converted into the toxic metabolite NAPQI, which is inactivated by a reaction with GSH. As a consequence, APAP-GSH and APAP-CYS metabolites can be detected in serum and the measurement of circulating APAP-CYS as a potential biomarker of paracetamol exposure has been described by different groups. At therapeutic non-hepatotoxic doses of APAP, APAP-CYS concentrations in serum will be lower than about 1.1 μM (Heard et al. 2016). When overdosed, relatively more NAPQI will be formed and it will not only react with GSH but also with cellular proteins, which will result in an increased amount of APAP-protein adducts and eventually necrosis. This leads to serum APAP-CYS levels up to 20–30 μM that remain elevated after APAP concentrations in serum have become unmeasurable (Curry et al. 2019).

As APAP-CYS is detected in serum also in the absence of liver damage, the conjugate is likely transported out of the hepatocytes into the plasma. In this study five ABC transport proteins that might be able to excrete APAP-CYS from cells were investigated for their role in the transport of APAP, APAP-GSH, and APAP-CYS. Whereas P-gp, BSEP, MRP3, MRP5, and BCRP did not transport any of the compounds, APAP-GSH appeared to be a substrate of MRP1, MRP2, and MRP4. MRP4 transported APAP-CYS and none of the ABC proteins were able to translocate APAP. This implicates that APAP-GSH can be effluxed into bile (MRP2) and plasma (MRP1 and MRP4), whereas APAP-CYS (MRP4) is transported to plasma. Although intracellular APAP-CYS concentrations are not known, the apparent affinity of 190 µM for MRP4 is relatively low as compared to reported serum levels. This may indicate that the transporter is not saturated up to APAP-CYS concentrations associated with toxic paracetamol dosages and that the efflux rate into plasma is also determined by the abundance of MRP4. This is particularly noteworthy as MRP4 expression is under normal conditions low, but can be upregulated in hepatocytes after exposure to paracetamol (Aleksunes et al. 2008a). Next to MRP4 induction, also MRP2, MRP5 and BCRP protein levels were increased in both APAP-treated rodents and APAP overdose patients (Aleksunes et al. 2005; Barnes et al. 2007; Ghanem et al. 2004; Gu and Manautou 2010). Nuclear receptors that have been shown to play a role in APAP toxicity can induce the expression of MRPs by activation of Nrf2 (Aleksunes et al. 2008b; Gu and Manautou 2010).

APAP-CYS is a potential biomarker for APAP-induced liver damage and might also be a valuable indicator of chronic APAP intoxication. MRP4 appears to be a hepatic transporter that determines the efflux of APAP-CYS into plasma. The characterization of this transport step is important for the development of APAP-CYS into a good biomarker, as its plasma concentration can be influenced by drug-transporter interactions and upregulation of MRP4.

## Electronic supplementary material

Below is the link to the electronic supplementary material.

Additional file 1: Fig. S1. Time-dependent transport of APAP-GSH or APAP-CYS by MRP1, MRP2 and MRP4. The vesicles incubated with 500 µM APAP conjugate during 2–20 min at 37 °C. Vesicles containing overexpressed eYFP were used as control (mock). Values are presented as mean ± SEM with *n* = 8.
